# Gray matter parcellation constrained full brain fiber bundling with diffusion tensor imaging

**DOI:** 10.1118/1.4811155

**Published:** 2013-07-01

**Authors:** Qing Xu, Adam W. Anderson, John C. Gore, Zhaohua Ding

**Affiliations:** ^1^ Vanderbilt University Institute of Imaging Science, Vanderbilt University, Nashville, Tennessee 37232‐2310 and Department of Electrical Engineering and Computer Science, Vanderbilt University, Nashville, Tennessee 37235‐1826; ^2^ Vanderbilt University Institute of Imaging Science, Vanderbilt University, Nashville, Tennessee 37232‐2310 and Department of Biomedical Engineering, Vanderbilt University, Nashville, Tennessee 37235‐1631; ^3^ Vanderbilt University Institute of Imaging Science, Vanderbilt University, Nashville, Tennessee 37232‐2310; Department of Electrical Engineering and Computer Science, Vanderbilt University, Nashville, Tennessee 37235‐1826; and Department of Biomedical Engineering, Vanderbilt University, Nashville, Tennessee 37235‐1631

**Keywords:** MRI: anatomic, functional, spectral, diffusion, Registration, Segmentation, biodiffusion, biomedical MRI, brain, image registration, image segmentation, medical image processing, pattern clustering, bundling, white matter fibers, diffusion tensor imaging, Involving electronic [emr] or nuclear [nmr] magnetic resonance, e.g. magnetic resonance imaging, Digital computing or data processing equipment or methods, specially adapted for specific applications, Image data processing or generation, in general, Brain, Medical imaging, Cluster analysis, Coherence, Diffusion, Tensor methods, Anatomy, Coherence imaging, Optical fiber testing, Image registration

## Abstract

****Purpose**:**

Studying white matter fibers from diffusion tensor imaging (DTI) often requires them to be grouped into bundles that correspond to coherent anatomic structures, particularly bundles that connect cortical/subcortical basic units. However, traditional fiber clustering algorithms usually generate bundles with poor anatomic correspondence as they do not incorporate brain anatomic information into the clustering process. On the other hand, image registration‐based bundling methods segment fiber bundles by referring to a coregistered atlas or template with prelabeled anatomic information, but these approaches suffer from the uncertainties introduced from misregistration and fiber tracking errors and thus the resulting bundles usually have poor coherence. In this work, a bundling algorithm is proposed to overcome the above issues.

****Methods**:**

The proposed algorithm combines clustering‐ and registration‐based approaches so that the bundle coherence and the consistency with brain anatomy are simultaneously achieved. Moreover, based on this framework, a groupwise fiber bundling method is further proposed to leverage a group of DTI data for reducing the effect of the uncertainties in a single DTI data set and improving cross‐subject bundle consistency.

****Results**:**

Using the Montreal Neurological Institute template, the proposed methods are applied to building a full brain bundle network that connects cortical/subcortical basic units. Based on several proposed metrics, the resulting bundles show promising bundle coherence and anatomic consistency as well as improved cross‐subject consistency for the groupwise bundling.

****Conclusions**:**

A fiber bundling algorithm has been proposed in this paper to cluster a set of whole brain fibers into coherent bundles that are consistent to the brain anatomy.

## I. INTRODUCTION

Magnetic resonance diffusion tensor imaging (DTI) has become a primary neuroimaging technique for noninvasive characterization of the structure and architecture of the human brain *in vivo*.^1^, ^2^ Since its first introduction in the early 1990s, this technique has been widely used to elucidate the structural basis for brain function both in healthy^3^, ^4^ and disease conditions.^5^, ^6^, ^7^, ^8^, ^9^, ^10^, ^11^ As alterations of brain function are necessarily accompanied by structural changes in the underlying neural circuits, characterizing these changes may offer valuable insights into the pathogenesis, extent or progression of the disease, thus holding the potential of guiding therapeutic interventions.

A large body of DTI‐based structural studies rests on fiber tractography,^12^ in which local tissue orientations depicted by DTI are combined and used to delineate and visualize the courses of fiber tracts at a macroscopic scale. Typically, individual fibers are first reconstructed, then grouped into coherent fiber bundles and assigned labels that have anatomic interpretations.^13^ Anatomically labeled fiber bundles provide the basis for quantitative analysis of fiber structures and comparisons of structural properties across different populations in a physiologically meaningful manner.

To group individual fibers into coherent and anatomically meaningful bundles, a plethora of fiber bundling methods have been proposed to date. These methods come in three different flavors: manual,^14^, ^15^, ^16^ clustering based,^17^, ^18^, ^19^, ^20^, ^21^, ^22^, ^23^, ^24^, ^25^ and knowledge‐based bundling.^26^, ^27^, ^28^, ^29^ Manual fiber bundling groups fibers using one or more regions of interest (ROIs) placed by an experienced operator as constraints to the fiber courses.^14^, ^15^, ^16^ Performance of the manual bundling hinges significantly on the accuracy of identifying anatomic landmarks or structural boundaries. Evidently, such a bundling method suffers from the drawback of low time efficiency, high operator biases, and poor intraoperator reproducibility. The situation is made worse by fuzzy or missing structural boundaries when inspected visually, which renders accurate and precise definitions of ROIs nontrivial.

The difficulties and limitations with manual bundling have been greatly ameliorated by automated fiber bundling, for which a rich literature of clustering algorithms has been developed in the recent past.^17^, ^18^, ^19^, ^20^, ^21^, ^22^, ^23^, ^24^, ^25^ With no need for prior ROI definitions, these methods group fibers automatically according to their similarities in geometrical properties such as shapes, locations, or other attributes. While the clustering based methods are able to generate coherent fiber bundles efficiently by means of fully automated algorithms, they are purely data driven, and hence bundle fibers without due regard to their anatomical plausibility.

A logical compromise between the manual‐ and clustering‐based bundling methods is knowledge‐based bundling.^13^, ^26^, ^27^, ^28^, ^29^ A common knowledge‐based method uses a template of prototypical fiber bundles that are constructed from a subset of data.^13^, ^28^ To bundle reconstructed fiber tracts for a given subject, the template is first transformed into the subject's native space via a registration procedure. All the fibers are then classified in reference to the prototypical bundles in the template. Because the prototypical bundles are usually defined manually, it would be pretty awkward to perform full brain fiber bundling, and quite problematic as well given the fact that our knowledge on distributions of fiber connection routes in the human brain is far from complete. A ramification of knowledge‐based methods is atlas‐based fiber bundling.^26^, ^27^ Instead of defining the courses of prototypical fiber bundles as above, the atlas based method typically bundles fibers with parcellated gray matter as constraints to fiber terminals. Of particular note, due to the availability of complete gray matter parcellations,^30^ the atlas‐based method has the inherent capability of bundling fibers of the entire brain automatically. A salient advantage of both the template‐ and atlas‐based methods is that expert knowledge is incorporated into the bundling process, and thus all resulting bundles in principle bear anatomical interpretations naturally. Meanwhile, these methods have largely alleviated the problems of low time efficiency and poor inter‐ and intraoperator reproducibilities encountered in manual bundling. It should be pointed out, however, that essential to the accuracy of the knowledge‐based methods is the performance of the registration algorithm used to transform the template or atlas into the subject's native space.

In our earlier work,^29^ we developed a unified fiber bundling and registration (UFIBER) algorithm using a template based approach. A set of well‐established fiber bundles were chosen to debut the process and performance of the algorithm. In the present study, we extend the UFIBER algorithm so that fibers that connect cortical/subcortical units in the entire brain can be bundled in a fully automatic manner and consistently across subjects. Similar to the work in Refs. 26 and 27, an atlas of gray matter parcellation defined in the standard MNI space is used.^30^ Obviously, using parcellated gray matter in the form of individual functional regions to constrain fiber bundling has direct benefits to integrated structural‐functional studies of the human brain.^31^ But unlike Refs. 26 and 27, which bundle fibers with some heuristic proximity rules, we pose the problem of full brain bundling as an optimization process cast with a more rigorous mathematical framework, in which constraints from gray matter region parcellation and coherence of white matter fiber bundling are simultaneously considered and optimized. This relaxes the requirements for accurate registration, which is often hard to satisfy if at all owing to practical difficulties such as poor image contrast between gray matter regions and fairly complex structures therein. Furthermore, consistent bundling across subjects offers great advantages in subsequent statistical analysis of fiber bundle properties as clinical studies are almost exclusively population based.

In the remainder of this paper, a gray matter constrained bundle model that integrates constraints from anatomical parcellation into a statistical fiber bundle model is first proposed in Sec. II. This is followed by a fiber bundling algorithm that generates both geometrically coherent and anatomically consistent bundles for a single subject in Sec. III. In Sec. IV, a groupwise bundling approach that improves cross‐subject bundling consistency is described. Evaluation of the proposed algorithm is presented in Sec. V. Finally, the main contributions of this work and potential future directions are discussed in Sec. VI.

## II. GRAY MATTER CONSTRAINED BUNDLE MODEL

Given a brain atlas, in which gray matter is fully partitioned into L disjoint ROIs and each ROI is associated with a label *l* = 1, 2, …, *L*, we assume a registration procedure transforms all the labeled ROIs into a subject's space using homomorphic mapping. Let **r** denote the coordinates of a terminal point of a fiber **x** in the subject's space, and *l*(***r***) denote the ROI label of **r**. Therefore, a fiber **x** can be represented by a space curve connecting *l*(**r1**) and *l*(**r2**), where **r1** and **r2** are the two terminals of fiber **x**, respectively.

### II.A. Uncertainties

The variables **r** and *l*(•) are observations of their true values, and thus contain uncertainty due to observational errors. Uncertainties of **r** arise from the fiber tracking procedure, which has been widely recognized as being highly susceptible to image noise.^32^, ^33^ Fundamentally, the noise susceptibility is attributable to the integrative nature of fiber tracking, which leads to greater errors and uncertainties toward the terminals of fiber tracts.^34^ Moreover, the commonly used echo‐planar imaging (EPI) sequences for DTI data acquisitions typically generate images with poor signal‐to‐noise ratio (SNR), creating a fair amount of uncertainty in the local fiber orientations estimated. Although measures can be taken to partly suppress the effect of noise prior to or during fiber tracking,^35^, ^36^, ^37^ the uncertainty from noise by nature always exists, albeit to a lesser extent after noise suppression. In addition, complex structures of white matter fibers at certain locations, along with inadequacies of the tensor model to capture them and the available fiber tracking algorithms, add another source of uncertainties to fiber tracts.^38^ Of note, complex fiber structures may, in principle, be resolved by using high angular resolution diffusion imaging (HARDi) (Refs. 39 and 40) or handled by probabilistic fiber tracking,^41^, ^42^, ^43^ but these techniques have not become routine utilities owing to other complications they involve.

Uncertainties of *l*(•) are caused by the fact that registration of brain magnetic resonance (MR) images is particularly error‐prone. As briefly alluded to before, there are two primary difficulties in the registration: (1) there is a virtual lack of intensity contrast between neighboring functional regions in the gray matter, which provides no intensity cues for registering structures therein; (2) structures in the gray matter are pretty complex and have considerable anatomical variations among individuals, rendering precise matching of structural details a daunting task if possible at all.^44^ Image noise and geometric distortions may contribute to the complexity of gray matter registration as well.

### II.B. Gray matter projection model

Given a subject, whose gray matter is parcellated into L disjoint, labeled regions via atlas mapping, the likelihood of a fiber terminal **r** belonging to ROI *l* = 1, 2, …, *L* is modeled with a Gaussian distribution as follows:

(1) $$\begin{array}{ccc} p(r|l) & = & {\left(2\pi {\sigma_{\text{ROI}}}^{2}\right)}^{-3/2}\\ & & \times \;\exp\left(-\frac{{\left(r-v\left(l,r\right)\right)}^{T}\left(r-v\left(l,r\right)\right)}{2{\sigma_{\text{ROI}}}^{2}}\right), \end{array}$$

where **v**(*l*, **r**) is a point in ROI *l* closest to **r** and T denotes the transpose operation on a vector.

In Eq. (1), distance is used in modeling of terminal‐to‐ROI probabilities, and the terminals of a fiber are projected into their closest points in cortical or subcortical gray matter regions. Variance σ_ROI_, which is isotropic in this model, is a parameter related to uncertainties in image registration and fiber tracking. It is set to a smaller value for more precise registration and tracking, reflecting the fact that terminal points are expected to lie closer to their true ROIs, and vice versa.

Brute‐force evaluation of the probability *p*(**r**|*l*) in Eq. (1) is computationally expensive. To improve time efficiency, a distance transform of ROI *l* is precomputed and stored in a function ϕ_*l*_(•). This avoids the time consuming process of searching in ROI *l* for the closest point to **r**. Therefore, *p*(**r**|*l*) can be evaluated as

(2) $$p\left(r|l\right)={\left(2\pi {\sigma_{\text{ROI}}}^{2}\right)}^{-3/2}\exp\left(-\frac{{\left(\phi_{l}\left(r\right)\right)}^{2}}{2{\sigma_{\text{ROI}}}^{2}}\right).$$

### II.C. Gray matter constrained bundle model

For efficient parameter estimation, fibers in a bundle are modeled with a Gaussian distribution, as in previous works.^22^, ^29^ With a further assumption of point independence in a fiber, the probability of a fiber **x** belonging to the bundle connecting *l*1 and *l*2 can be expressed as

(3) $$\begin{array}{ccc} p(x|\mu_{l1,l2},\sigma_{l1,l2}) & = & \prod_{i=1}^{m}{\left(2\pi {\sigma_{l1,l2}}^{2}\right)}^{-3/2}\\ & & \times \;\exp\left(-\frac{{\left(x_{i}-\mu_{l1,l2,i}\right)}^{T}\left(x_{i}-\mu_{l1,l2,i}\right)}{2\sigma_{l1,l2}^{2}}\right), \end{array}$$

where i indexes points along the fiber **x** and **μ**_*l*1,*l*2_ is the medial axis of the bundle connecting *l*1 and *l*2. Typically, **μ**_*l*1,*l*2_ is an unknown parameter that needs to be estimated by an optimization scheme. Here, an isotropic variance σ_*l*1,*l*2_ is used for all points along the fibers in all bundles (denoted as σ_bundle_), as some bundles may contain a small number of fibers, which makes estimation of point‐specific covariance and its inverse computationally unstable. **x** and **μ**_*l*1,*l*2_ are resampled to an equal number of points and **x**_*i*_ and **μ**_*l*1,*l*2,*i*_ denote their *i*th point.

When clustering fibers solely based on this Gaussian model, fibers with similar spatial courses would be grouped into a bundle without respect to their terminal locations. To use gray matter parcellation as constraints to fiber terminals, the projection model of fiber terminal points can be included. Thus, for a fiber **x** with terminal points **r1** and **r2**, the likelihood of **x** being in the bundle connecting *l*1 and *l*2 is defined as the joint probability of fiber distribution in a bundle and gray matter parcellation distribution, i.e.,

(4) $$\begin{array}{ccc} p(x|l1,l2) & = & p(x|\mu_{l1,l2},\sigma_{\text{bundle}},\sigma_{\text{ROI}})\\ & = & p\left(x|\mu_{l1,l2},\sigma_{\text{bundle}}\right)p\left(r1,r2|l1,l2\right), \end{array}$$

where

p(r1,r2|l1,l2)=max(p(r1|l1)p(r2|l2),p(r1|l2)p(r2|l1)).

Note that the correspondence between **r1**, **r2**, and *l*1, *l*2 is chosen to be the one yielding a greater overall probability of terminal assignments evaluated using Eq. (2).

According to the model in Eq. (4), fibers are bundled considering both the coherence of fiber tracts and proximity of their terminals to designated ROIs. Trade‐offs between the fiber bundle coherence and terminal proximity are regulated by relative magnitudes of the values of bundle variance σ_bundle_ and ROI variance σ_ROI_.

## III. GRAY MATTER PARCELLATION CONSTRAINED FIBER BUNDLING FOR A SINGLE SUBJECT

In this section, an algorithm is proposed to bundle the entire brain of a single subject based on the gray matter constrained bundle model.

### III.A. Objective function

With a complete gray matter parcellation scheme, fibers of the entire brain can be modeled as a mixture of gray matter constrained bundle models

(5) $$\begin{array}{ccc} & & p(x_{\text{all}}|\mu ,\sigma_{\text{bundle}},\sigma_{\text{ROI}})\\ & & \;=\prod_{j=1}^{J}\sum_{l2=l1+1}^{L}\sum_{l1=1}^{L}p(x_{j}|\mu_{l1,l2},\sigma_{\text{bundle}},\sigma_{\text{ROI}}), \end{array}$$

where *j* indexes a fiber in the set of *J* fibers over the entire brain and σ_bundle_, σ_ROI_ are, respectively, the bundle and ROI variance, which in this work are fixed based on several investigation studies (see Sec. V D below). Mixture proportions are set to one for all the bundles. This is because it is observed that the number of fibers in some bundles, such as those passing the corpus callosum, is significantly greater than that of other bundles and it is not desirable that fibers prefer to be assigned to bundles with big mixture proportions. Therefore, the only variable that needs to be estimated is the bundle medial axis μ_*l*1,*l*2_. Note that the second summation is taken from *l*1 + 1 to *L* for the index *l*2 since we assume μ_*l*1,*l*1_ does not exist and μ_*l*2, *l*1_ and μ_*l*1,*l*2_ are identical.

Assuming each fiber is an independent sample from this distribution, an optimal **μ** can be estimated by maximizing the following likelihood:

(6) $$\begin{array}{ccc} \hat{\mu } & = & \arg\max_{\mu }p\left(x_{\text{all}}|\mu ,\sigma_{\text{bundle}},\sigma_{\text{ROI}}\right)\\ & = & \arg\max_{\mu }\prod_{j=1}^{J}\sum_{l2=l1+1}^{L}\sum_{l1=1}^{L}p\left(x_{j}|\mu_{l1,l2},\sigma_{\text{bundle}},\sigma_{\text{ROI}}\right). \end{array}$$

### III.B. Expectation and maximization (EM) algorithm

To solve for an optimal **μ** in Eq. (6), the classic solution, EM algorithm,^45^ is employed in this work. Given an initial **μ** ^0^, an expectation (E), step and maximization (M) step are alternatively performed until convergence. In the E step, based on the current estimation of **μ** ^*n*−1^, the fiber‐to‐bundle membership is computed with the formula below

(7) $$m_{j,(l1,l2)}^{n}=\frac{p\left(x_{j}|\mu_{l1,l2}^{n-1},\sigma_{\text{bundle}},\sigma_{\text{ROI}}\right)}{\sum_{l2=l1+1}^{L}\sum_{l1=1}^{L}p\left(x_{j}|\mu_{l1,l2}^{n-1},\sigma_{\text{bundlle}},\sigma_{\text{ROI}}\right)},$$

where $m_{j,(l1,l2)}^{n}$ represents the membership of fiber **x**_*j*_ to bundle (*l*1, *l*2). In essence, $m_{j,(l1,l2)}^{n}$ is the likelihood of fiber **x**_*j*_ to bundle (*l*1, *l*2) normalized across all the bundles.^46^, ^47^

In the M step, using the estimated $m_{j,(l1,l2)}^{n}$, an optimal **μ** ^*n*^ can be found by maximizing the likelihood below:

(8a) $$\begin{array}{ccc} E(\mu^{n}) & = & \sum_{j=1}^{J}\sum_{l2=l1+1}^{L}\sum_{l1=1}^{L}m_{j,(l1,l2)}^{n}\\ & & \times \log\left(p\left(x_{j}|\mu_{l1,l2}^{n},\sigma_{\text{bundle}},\sigma_{\text{ROI}}\right)\right), \end{array}$$

which leads to the update function for **μ** ^*n*^,

(8b) $$\mu_{l1,l2}^{n}=\frac{\sum_{j=1}^{J}m_{j,(l1,l2)}^{n}x_{j}}{\sum_{j=1}^{J}m_{j,(l1,l2)}^{n}}.$$

The above update scheme is essentially a weighted sum of all fibers using the membership. Using only the Gaussian bundle model [Eq. (3)], the membership is solely determined by the distance between individual fibers and their corresponding bundle medial axis, which leads to a minimization of the inbundle variation or the coherence of resulting bundles. Using the gray matter constrained bundle model, a decay term [Eq. (1)] is used to attenuate the membership based on the distances between fiber terminal points to the corresponding ROIs, which constrains the bundling process so that the bundle center would not deviate too much from its corresponding ROI. The inherent coherence minimization force is to correct inaccurate ROI labeling caused by image misregistration, while the parcellation constraint places a certain level of confidence on coregistrated ROIs.

### III.C. Implementation issues

From Eqs. (7) and (8), it can be seen that the computational complexity is proportional to the total number of fibers, which is huge (∼40 000) for each subject. To reduce the number of fibers in the computation, *m*_*j*, (*l*1,*l*2)_ is set to zero if one of**x**_*j*_'s end points has a distance greater than 3σ_ROI_ from ROI *l*1 or *l*2. Then *m*_*j*, (*l*1,*l*2)_ would never be evaluated, nor would **x**_*j*_ be involved in the update of μ_*l*1,*l*2_. With this simplification, a significant amount of computation is avoided, leading to a CPU time of about 1 min per iteration (Intel Xeon 5150 2.66 GHz).

To find an initial estimate of **μ** ^0^, all fibers are first classified into a bundle that connects a pair of ROIs that their end points are closest to. Then **μ** ^0^ for each pair of ROIs can be computed as the mean value of all the fibers in the bundle connecting them. Since all fibers have already been resampled to the same number of points, each point in the mean fiber is simply the average point of all the corresponding points in the bundle.

In each E step, fibers are assigned to the bundle with the maximum membership value. The algorithm automatically terminates when the total number of changes to fiber‐to‐bundle assignment or the number of iterations reaches a preset threshold (20 and 10, respectively, in this work).

## IV. GROUP CONSISTENT FIBER BUNDLING

In this section, an algorithm is proposed to bundle the entire brains for a group of subjects based on the gray matter constrained bundle model.

### IV.A. Objective function

The proposed groupwise bundling is formulated as estimating a common bundle model for a given set of subjects. Each fiber set is assumed to be an independent sample from the underlying common bundle model. Such an assumption can be safely made when fiber sets are from a group of subjects with the same condition or even from the same subject. Let ***x*** ^*s*^ denote the *s*th fiber set in the subject group, where *s* could range from 1 to *S*, the total number of subjects in the group. Using the gray matter projection bundle model, the unknown parameter, bundle centroids **μ**, can be estimated using the below Bayesian rule,

$$\hat{\mu }=\arg\max_{\mu }\prod_{s=1}^{S}p\left({x_{\text{all}}}^{s}|\mu ,\sigma_{\text{bundle}},\sigma_{\text{ROI}}\right),$$

where σ_bundle_, σ_ROI_ are the bundle variance and ROI variance, respectively.

One problem with the above estimation is that each fiber set is in its own native space as subjects may be scanned in different positions in scanners. Therefore, it is necessary to transform all fiber sets into a common space, where the bundle model will be estimated. A T1 or fractional anisotropy (FA) based image registration procedure may be applied to this problem, but these approaches align fibers by only minimizing their image intensity difference without considering the underlying fiber directions. As T1 or FA intensity only indicates the type of the underlying tissue but nothing about fiber orientations, two voxels with different fiber orientations may be incorrectly considered to correspond simply due to their T1 or FA intensity similarity. To address this issue, fibers shall be utilized to make the alignment among a group of subjects. To avoid the alignment of whole fiber sets, which contain a huge number of fibers, bundle centroids can be efficiently aligned with each other through a nonrigid transformation. However, since the fiber bundles are yet to be estimated, there are no reliable bundles that can be used to make this alignment.

As the solutions to the groupwise bundling and spatial alignment could benefit each other, these two problems are coupled into a unified objective function and optimal transformations and bundling are jointly estimated. Let ***T*** ^*s*^ be a transformation that warps the fiber set ***x*** ^*s*^ into a common space. This can be cast as an optimization problem that simultaneously seeks an optimal model **μ** and optimal transformations ***T*** ^*s*^ from subject fiber sets’ native spaces to the common space given a group of fiber sets ***x*** ^*s*^. Using a Bayesian estimation framework, an optimal solution can be obtained by a maximum *a posteriori* (MAP) approach,

(9) $$\begin{array}{ccc} \theta & = & \left(T^{1,2,\ldots ,S}\mu \right)\\ & = & \arg\max_{T^{1,2,\ldots ,S}\mu }\prod_{s=1}^{S}p\left({x_{\text{all}}}^{s}|\mu ,\sigma_{\text{bundle}},\sigma_{\text{ROI}},T^{s}\right). \end{array}$$

To derive the expression of $p({x_{\text{all}}}^{s}|T^{1,2,\ldots S},\mu ,\sigma_{\text{bundle}},\sigma_{\text{ROI}})$, it is assumed that each transformed fiber in the common space is an independent and identically distributed sample that is drawn from the distribution of the common bundle model, which leads to the below formula,

(10) $$\begin{array}{ccc} & & \prod_{s=1}^{S}p({x_{\text{all}}}^{s}|T^{s},\mu ,\;\sigma_{\text{bundle}},\sigma_{\text{ROI}})\\ & & \;=\prod_{s=1}^{S}p(T^{s}({x_{\text{all}}}^{s})|\mu ,\sigma_{\text{bundle}},\sigma_{\text{ROI}})\\ & & \;=\prod_{s=1}^{S}\prod_{j=1}^{M^{s}}p(T^{s}\left({x^{s}}_{j}\right)|\mu ,\sigma_{\text{bundle}},\sigma_{\text{ROI}})\\ & & \;=\prod_{s=1}^{S}\prod_{j=1}^{M^{s}}\sum_{k=1}^{K}p(T^{s}\left({x^{s}}_{j}\right)|\mu_{k},\sigma_{\text{bundle}},\sigma_{\text{ROI}}), \end{array}$$

where *j*, *k*, *s* index fibers in a target fiber set, fiber bundles in the common bundle set, and subject in the group, respectively. There are totally ***K*** bundles that need to be estimated and ***M*** ^*s*^ fibers for each subject fiber set **x** ^*s*^. $p(T^{s}\left(x_{j}^{s}\right)|\mu_{k},\sigma_{\text{bundle}},\sigma_{\text{ROI}})$ is evaluated using the same formula as Eq. (4), where a bundle is indexed by the ROI pair (*l*1, *l*2). The index *k* is in essence the same as the (*l*1, *l*2). The optimal parameters (**T** ^1, 2, …, *S*^,**μ**) can be found by maximizing the above probability [Eq. (10)].

### IV.B. EM algorithm

The above optimization problem can be solved with the Expectation and Maximization algorithm. Let *n* denote the iteration of the EM algorithm and (***T*** ^*n*, 1, 2, …, *S*^,**μ** ^*n*^) denote the resulting parameters estimated in that iteration. In the E step, the membership probability of a fiber $x_{j}^{s}$ to the *k*th bundle is estimated as follows:

(11) $$m_{j,k}^{s,n}=\frac{p\left(T^{n-1,s}\left(x_{j}^{s}\right)|\mu_{k}^{n-1},\sigma_{\text{bundle}},\sigma_{\text{ROI}}\right)}{\sum_{k=1}^{K}p\left(T^{n-1,s}\left(x_{j}^{s}\right)|\mu_{k}^{n-1},\sigma_{\text{bundle}},\sigma_{\text{ROI}}\right)}.$$

In the M step, based on the fiber membership $m_{j,k}^{s,n}$, the original objective likelihood is turned into

(12) $$\begin{array}{ccc} E_{\text{EM}}\left(T^{n,1,2,\ldots S},\mu^{n}\right) & = & \sum_{s=1}^{S}\sum_{j=1}^{M^{s}}\sum_{k=1}^{K}m_{j,k}^{s,n}\\ & & \times \log\left(p\left(T^{n,s}\left(x_{j}^{s}\right)|\mu_{k}^{n},\sigma_{\text{bundle}},\sigma_{\text{ROI}}\right)\right). \end{array}$$

The above objective function can be optimized by firstly fixing the transformations **T** ^*n*, 1, 2, …*S*^ to be **T** ^*n* − 1, 1, 2, …*S*^and then solving the differential equations,

$$\frac{dE_{\text{EM}}}{d\mu^{n}}=0,$$

which leads to the below solution

(13) $${\mu_{k,i}}^{n}=\frac{\sum_{s=1}^{S}\sum_{j=1}^{M^{s}}m_{j,k}^{s,n}T^{s,n-1}\left(x_{j,i}^{s}\right)}{\sum_{k=1}^{K}\sum_{s=1}^{S}\sum_{j=1}^{M^{s}}m_{j,k}^{s,n}},$$

where *i* is used to index the points on the fiber.

After ${\mu_{k,i}}^{n}$ is computed and fixed, we minimize the below objective function to estimate transformations **T** ^*n*, 1, 2, …*S*^,

$$\begin{array}{ccc} E_{\text{EM}}\left(T^{n,1,2,\ldots S}\right) & = & \sum_{s=1}^{S}\sum_{j=1}^{M^{s}}\sum_{k=1}^{K}m_{j,k}^{s,n}\sum_{i=1}^{N_{k}}\left(T^{s,n}\left(x_{j,i}^{s}\right)-\mu_{k,i}^{n}\right)\\ & & \times {\left(T^{s,n}\left(x_{j,i}^{s}\right)-\mu_{k,i}^{n}\right)}^{T})), \end{array}$$

where *N*_*k*_ is the number of fibers in the *k*th subject.

The minimization of *E*_EM_(**T** ^*n*, 1, 2, …*S*^) is actually a least‐squares problem, the computational cost of which depends mainly on the number of target fibers *M* ^*s*^ in each subject and the total number of subjects *S*. To improve the computation efficiency, we circumvent the direct optimization of *E*_EM_(**T** ^*n*, 1, 2, …*S*^) by minimizing a simpler form *E*_EM_′(**T** ^*n*, 1, 2, …*S*^) as follows:

(14) $$\begin{array}{ccc} E_{\text{EM}}\prime \left(T^{n,1,2,\ldots S}\right) & = & \sum_{k=1}^{K}\sum_{i=1}^{N_{k}}\left(T^{s,n}\left(y_{k,i}^{n}\right)-\mu_{k,i}^{n}\right)\\ & & \times {\left(T^{s,n}\left(y_{k,i}^{n}\right)-\mu_{k,i}^{n}\right)}^{T})), \end{array}$$

where

$$y_{k,i}^{n}=\sum_{s=1}^{S}\sum_{j=1}^{M^{s}}m_{j,k}^{s,n}x_{j,i}^{s}.$$

Here, $y_{k,i}^{n}$ can be interpreted as the current estimation of bundle centroids for individual subject *k*. The optimization of Eq. (14) is essentially aligning bundle centroids of each subject to their common model centroids. This simplification would make the computational complexity proportional to the number of bundles and the number of points in each bundle, which is much smaller than the total number of fibers in the whole data set. In the case of whole brain bundling, even with reduction, the computational complexity is still unacceptable due to a large number of bundles (typically ∼1000). Therefore, each centroid is further downsampled (three times) to fit the computation to our hardware resources.

Theoretically, any form of transformation can be used in the above registration framework. The thin‐plate spline (TPS) transformation is chosen in this work due to its smoothness in deformation fields and closed‐form solution for warping and parameter estimation.^42^

To compute the initial values for the parameters (**T** ^0,1,2, …, *S*^,**μ** ^0^), a rigid registration is first performed to give a rough alignment of all the subjects to the template (**T** ^0,1,2, …, *S*^). Then fibers from all subjects are transformed to the template space and classified as bundles that connect a pair of ROIs. **μ** ^0^ is obtained by combining the same bundles from all the subjects and computing their mean values.

## V. EXPERIMENTS AND RESULTS

### V.A. Gray matter parcellation template

In Tzourio‐Mazoyer's work,^18^ the gray matter in the Montreal Neurological Institute (MNI) single subject MRI data is manually labeled into 90 ROIs, the so‐called automated anatomical labeling (AAL) mask, including 39 cortical regions on each brain hemisphere and 12 subcortical regions. Similar to the work of Gong *et al.*,^2^ these ROIs are considered to be basic units of the brain gray matter, and fiber bundles connecting pairs of ROIs are studied in this work.

### V.B. Imaging and fiber tracking

The T1 weighted and diffusion weighted images were acquired for ten healthy human subjects using a 3T Philips Achieva MR scanner. Informed consent was given by the subject according to a protocol that was approved by the local ethics committee. Each T1 volume contains a 170 × 256 × 256 matrix with an isotropic resolution of 1 × 1 × 1 mm^3^. The DWI data were acquired with 32 noncollinear weighting directions and a single shot, echo‐planar, pulsed gradient spin echo imaging sequence with a diffusion weighting factor (i.e., b‐value) of 1000, which generated a volume of 128 × 128 × 60 voxels at an isotropic resolution of 2 × 2 × 2 mm^3^ for each direction. Three repeated scans were performed and coregistered and corrected for motion and distortion. A linear least‐squares fitting was used to estimate diffusion tensors. Then a streamline tracking algorithm was started at all voxels with FA > 0.15, and followed sequentially along the local principal diffusion direction at a step size of 2 mm. A fiber was terminated when voxels with FA below 0.15 were met or the angle between the principal diffusion directions of two consecutive points exceeded 41°. The above procedure generated a whole volume fiber set (∼40 000 fibers) for each subject.

### V.C. Registration of AAL mask with fibers

To transform a subject's fibers to the MNI space, the MNI T1 template is registered with the subject's T1 images using intensity difference as a metric. As the subject's T1 images are not in the exact space as the DTI data and fibers, the T1 images need to be first aligned with the associated b0 diffusion weighted images. As the two types of images have different intensity distributions, normalized mutual information^42^ is used as the registration metric. Moreover, a rigid transformation is estimated since both image sets are from the same subject and thus a rigid transformation should be sufficient to characterize the transformation. The T1 images are warped and then registered with the T1 MNI template. The resulting transformation maps fibers from DTI space to MNI space, where the AAL mask is used as the parcellation constraint.

### V.D. Evaluations of single‐subject based bundling

Although an anatomical mapping is already provided by the above registration process, the inaccuracy of registration likely results in bundles with poor coherence. On the other hand, a coherent bundle from a clustering algorithm may have poor consistency with this anatomical mapping.

#### V.D.1. Metrics

To quantitatively characterize the bundling results, two metrics are proposed in this work: (1) mean inbundle variation and (2) mean end‐to‐ROI distance. Mean inbundle variation (MIV) generally measures the coherence of a bundle, which is expressed as the mean distance of fibers to their corresponding bundle centroids,

(15) $$\text{MIV}=\frac{1}{J}\sum_{j=1}^{J}|x_{j}-\mu_{\varphi (j)}|,$$

where |•| denotes the distance between two fibers and φ(•) is the fiber assignment function that maps fiber **x**_*j*_ to the bundle with maximum membership. The total distances are normalized by the number of fibers *J*. The mean end‐to‐ROI distances (MED) characterize the deviation of bundles from their assigned ROI pair. This metric can be expressed as follows:

(16) $$\text{MED}=\frac{1}{J}\sum_{j=1}^{J}\phi_{l1_{\varphi (j)}}\left({r1}_{j}\right)+\phi_{l2_{\varphi (j)}}\left(r2_{j}\right),$$

where **r1**_*j*_ and **r2**_*j*_ are start and end points of fiber **x**_*j*_, and *l*1_φ(*j*)_ and *l*2_φ(*j*)_ correspond to the two ROIs, which define the bundle that **x**_*j*_ is assigned to. A smaller MED usually suggests resulting bundles are consistent with the original ROI definition, while a bigger MED shows resulting bundles may not connect their corresponding ROIs correctly.

#### V.D.2. Convergence

**Figure 1:**
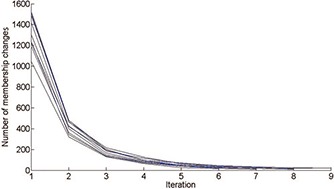
The variation of the number of fiber‐to‐bundle assignment changes with respect to iteration index for all ten subjects.

The single‐subject bundling algorithm (σ_bundle_ = 2 voxels, σ_ROI_ = 2 voxels) is applied to all ten subjects’ DTI data. The number of fiber assignment changes is recorded at each iteration, and the algorithm achieves convergence (below 20 changes) in nine iterations for all ten cases (see Fig. 1).

#### V.D.3. Effect of the ROI variance

**Figure 2:**
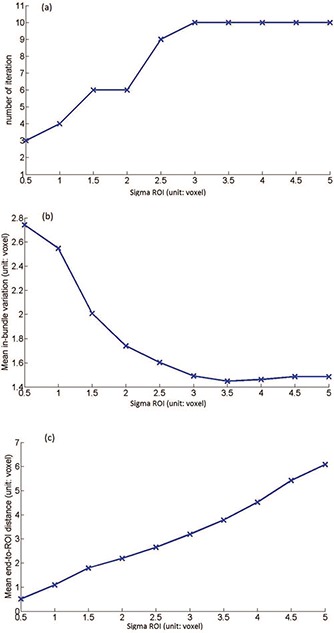
The variations of (a) number of iterations to convergence, (b) mean inbundle variation, and (c) mean end‐to‐ROI distance with respect to different ROI variance.

For one of the subjects, the proposed bundling algorithm is applied several times with variance σ_ROI_ changing from 0.5 to 5. The plot in Fig. 2(a) shows that the number of iterations that is needed to reach convergence increases with the increase of σ_ROI_, which indicates that a smaller σ_ROI_ would make the algorithm converge faster. The increase of variance puts less confidence on the ROI mapping and thus the effect of the ROI constraint is weakened so that the clustering process takes longer. In Fig. 2(b), the mean inbundle variation decreases with the increase of σ_ROI_, which leads to a decreasing contribution of end point positions to the bundle model. It also can be seen from Fig. 2(c) that the mean end‐to‐ROI distances are increasing with the increase of σ_ROI_, as a bigger σ_ROI_ would reduce the “force” of dragging a bundle to its corresponding ROI pair so that the bundle is more free to move away from ROIs. From these results, we can see that a zero σ_ROI_ would turn the algorithm into a simple one that just labels fibers based on their closest ROI pairs, while a positive infinite σ_ROI_ would turn the algorithm into a pure clustering algorithm without consideration of the anatomical information. Based on this result, σ_ROI_ is set to 2 for the following experiments as this setting can generate relatively smaller values for both the mean inbundle variance and end‐to‐ROI distances.

#### V.D.4. Comparisons with baseline methods

In this experiment, the proposed algorithm is compared with two other baseline methods: (1) a clustering without gray matter projection model and (2) directly assigning fibers based on their closest ROI label.

**Figure 3:**
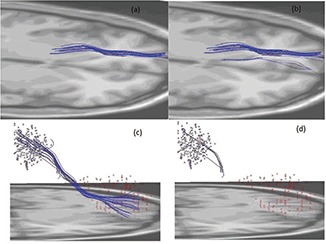
An illustrative example of differences of the parcellation constrained bundling with the clustering only and the parcellation constraint only method. (a) The bundle connecting the triangular part of the left inferior frontal gyrus to the left caudate (parcellation constrained clustering). (b) The same bundle as (a) (parcellation constraint only). (c) The bundle connecting the left hippocampus to the left supplementary motor area (parcellation constrained clustering). (d) The same bundle as (c) (clustering only).

Two examples of the resulting bundles from these methods are illustrated in Fig. 3. In the first row, the bundle connecting the triangular part of the left inferior frontal gyrus to the left caudate is displayed for both the proposed method Fig. 3(a) and the ROI constraint only method Fig. 3(b). It can be seen that in Fig. 3(b) several outlier fibers, which deviate significantly from the majority of the bundle, are also grouped into this bundle as their end points fall into the corresponding ROIs. These fibers are discarded in the proposed method as their existence in this bundle would increase the inbundle variation or reduce the coherence of the bundle. On the other hand, a clustering algorithm that only aims at minimizing such coherence will also produce some erroneous results as in Fig. 3(d). Although initialized as a bundle connecting the left hippocampus to the left supplementary motor area, the bundle still finally converges to a very small bundle that does not even connect these two specific ROIs. The proposed algorithm is capable of constraining the bundle to the two ROIs so that the resulting bundle will not move too far away.

**Figure 4:**
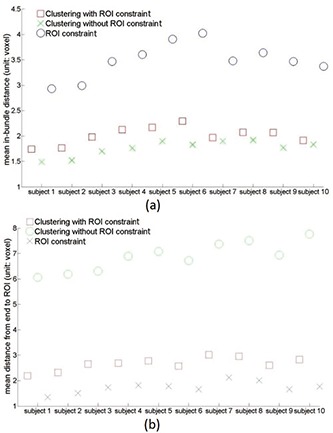
The plot of mean inbundle variation (a) and end‐to‐ROI distances (b) for the parcellation constrained bundling and the two baseline methods.

To further quantify this performance, the mean inbundle variation and end‐to‐ROI distances are summarized in Fig. 4 for all ten subjects. Although the clustering only and ROI constrained only methods achieved minimum inbundle distance and end‐to‐ROI distances, respectively, they also generate large values for the other metric. On the other hand, the proposed algorithm yields close‐to‐minimum values for both metrics without deteriorating the other metric.

#### V.D.5. Demonstrations of resulting bundles

**Figure 5:**
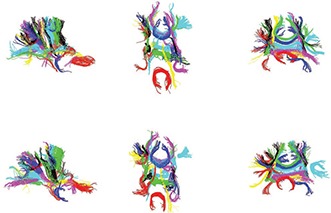
Saggittal (left), axial (middle), and coronal (right) view of whole brain bundling results for two example subjects (subjects 3 and 6) using the parcellation constrained bundling.

Due to the image misregistration and the uncertainty in the DTI fibers, it is possible that some bundles are generated for nonexisting connections between two cortical/subcortical regions. To eliminate these outlier bundles, only bundles consistent across the whole group are identified and kept as valid connections. To measure the consistency, we computed the mean differences of each individual bundle's mean and its group mean (discussed further in Sec. V E). Bundles with above 2.5 voxels difference are discarded, resulting in a total of 36 bundles which are rendered in Fig. 5 for two example subjects.

### V.E. Evaluations of groupwise bundling

In the case of the groupwise bundling, the initial registration is not considered to be sufficient to provide bundlewise alignment. Therefore, in addition to bundling, bundles from different subjects are aligned simultaneously, which contributes to the preservation of cross‐subject consistency.

#### V.E.1. Metrics

To measure the cross‐subject bundle consistency, a mean bundle centroid difference (MBCD) is proposed and computed as follows:

(17) $$\text{MBCD}=\frac{1}{\text{KS}}\sum_{k=1}^{K}\sum_{s=1}^{S}\left|\mu_{k}^{s}-\mu_{k}\right|,$$

where μ^*s*^ is the subject‐specific bundle centroid, μ is the bundle centroid of the group, and *k* indexes the bundle. This metric essentially measures the deviation of subject‐specific models from the group mean. The groupwise mean inbundle variation (GMIV) is also measured by computing the average distance of fibers to their corresponding group bundle centroids

(18) $$\text{GMIV}=\frac{1}{\sum_{s=1}^{S}M^{s}}\sum_{s=1}^{S}\sum_{j=1}^{M^{s}}\left|x_{j}^{s}-\mu_{\varphi (x_{j}^{s})}\right|,$$

where the assignment function $\varphi (x_{j}^{s})$ assigns the fiber $x_{j}^{s}$ to a group common bundle. The coherence metric GMIV is related to consistency, as consistent bundling tends to yield coherent bundles.

#### V.E.2. Baseline methods

To demonstrate the main advantages of the proposed algorithm, we compared it with three different baseline methods. In method I each fiber set from the subject group is individually bundled using the gray matter parcellation constrained bundling algorithm with no attempt to correct for image misregistration. Method II bundles the fibers in a way identical to method I except that a TPS transformation is applied afterwards to transform each individual subject's bundle centroids to the corresponding common centroids. Method III uses a joint clustering scheme that treats fibers from all subjects as a single fiber set, and performs gray matter parcellation constrained bundling on this combined data set. Note that all fibers are transformed to the MNI space by the preliminary image coregistration for all the three methods.

The above metrics are computed and summarized in Table I for all four methods, including the proposed method and the other three baseline methods. It can be seen that the proposed consistent groupwise bundling algorithm has the smallest values for both metrics, which indicates its superiority in preserving consistency. Although a nonrigid transformation is also used in method II to align bundles with their group mean, the resulting metric values still cannot compete with the proposed algorithm due to the absence of this transformation in the bundling process. In each iteration of the proposed algorithm, the clustering would favor the direction that could generate more consistent bundling. On the other hand, the joint clustering without transformation is less likely to reduce consistency very much due to the misalignment caused by scalar‐image coregistration. Therefore, from this example, one can see that it is important to integrate the nonrigid transformation into the clustering process.

**Table I:** The consistency and coherence metrics (in voxels) for all four types of groupwise bundling methods.

	Group consistent fiber bundling	Method I	Method II	Method III
GMIV	3.74	4.98	4.54	4.41
MBCD	1.31	2.78	2.20	1.90

#### V.E.3. Demonstrations of resulting bundles

**Figure 6:**
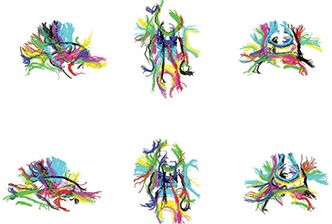
Saggittal (left), axial (middle), and coronal (right) view of whole brain bundling results for two example subjects (subjects 3 and 6) using the groupwise bundling.

To demonstrate some identified connections, the same elimination procedure as used for the single subject bundling experiments (see Sec. ???) is applied to the resulting fiber bundles, generating 45 bundles for each subject (see Fig. 6 for two representative subjects).

## VI. CONCLUSION AND DISCUSSION

To obtain a set of DTI fiber bundles, one must start with DTI imaging, track fibers by connecting DTI tensor directions, and then cluster these fibers based on fiber similarities. There is a significant amount of noise and uncertainty in each step. Noise introduced in the DTI imaging step makes the estimated fiber direction at each voxel unreliable. By following these unreliable directions a fiber is generated. Therefore, any uncertainty in these directions causes the fiber to vary from its true shape and position. This issue is more serious on the ends of the fiber as these points are farther away from the starting point and errors are accumulated in the tracking process. To cluster these fibers into bundles, fiber similarity needs to be measured. However, this step is more challenging, as it is still an open question on how to measure curve similarity and there are lots of issues such as point correspondence and metric and so on. Therefore, this kind of noise must be reduced to generate accurate fiber bundles for a study.

Using prior knowledge and smoothing is the major way of reducing noise. For example, on DTI images it could be assumed that intensities of close voxels should be similar. This prior knowledge can be utilized to smooth images by averaging intensities of neighboring voxels to get rid of imaging noise. If it is only assumed that only voxels in homogenous regions have similar intensities, one should only smooth voxels on nonboundary regions, which leads to an anisotropic denoising method. Different prior knowledge could lead to different denoising algorithms. Similarly in fiber tracking, the prior knowledge that two neighboring points along the curve should have similar directions has been frequently used to reduce the noise in the fiber tracking, although this prior can be integrated into fiber tracking in different ways, such as simply smoothing a curve to reduce curvature or using it as a Bayesian prior.

The basic motivation of this paper is to introduce useful priors to reduce the noise in the fiber bundling process. In addition to the traditional prior that similar fibers should stay in the same bundle, we proposed a novel prior based on the anatomical information of human brain, i.e., bundles should have meaningful correspondence to the real brain anatomy. The anatomical prior is much more reliable than the imaging data obtained through a single DTI scan, as it is built from numerous imaging data sets and validated by human experts. Therefore, fiber bundles deviating from the anatomy structure are corrected by enforcing this prior. We also developed a Bayesian algorithm to combine the traditional and the anatomical prior to make them work together. Another useful prior introduced into fiber bundling is that bundles from a group of similar subjects should have similar structures. With this prior, bundles in a subject are smoothed by bundles from his/her “neighboring” subjects. A groupwise bundling algorithm is proposed in this paper to make this prior integrated into the Bayesian clustering framework. In summary, the major contribution of this paper is the exploring of different priors to reduce the uncertainty of DTI fiber bundling.

Similar to other smoothing techniques, oversmoothing could be a potential issue to the use of priors in reducing noise. In other words, true individual variations could also be falsely treated as noise and removed due to its difference from the prior, such as the brain anatomical template or the mean bundle of the subject group. This could be the focus of future work, identifying the right procedure of tuning the algorithm parameters.

Two important aspects ignored in this paper are the underlying diffusion models and fiber tracking algorithms that are used to generate the input to the presented method. Although they are not the focus of this paper, we do realize that the resulting fiber bundles are heavily affected by these factors. The proposed method can only yield the fiber bundles for the entire brain only if the input fibers contains all the fibers in the entire brain. We use a tensor model and streamline fiber tracking algorithm for demonstration purposes. However, more accurate models with higher angular resolutions and more accurate fiber tracking algorithms (e.g., probabilistic tracking algorithms) could be potentially used to improve the coverage and accuracy of the resulting fiber bundles.
